# Mechanism Analysis of Metabolic Fatty Liver on Largemouth Bass (*Micropterus salmoides*) Based on Integrated Lipidomics and Proteomics

**DOI:** 10.3390/metabo12080759

**Published:** 2022-08-17

**Authors:** Moyong Xue, Ting Yao, Min Xue, Frédéric Francis, Yuchang Qin, Ming Jia, Junguo Li, Xu Gu

**Affiliations:** 1 Feed Research Institute, Chinese Academy of Agricultural Science, Beijing 100081, China; 2 Functional & Evolutionary Entomology, Agro-Bio-Tech Gembloux, University of Liege, 5030 Gembloux, Belgium; 3 Institute of Animal Science, Chinese Academy of Agriculture Sciences, Beijing 100193, China; 4 Beijing Institute of Feed Control, Beijing 110108, China

**Keywords:** metabolic liver disease, integrated analysis, proteomics, lipidomics, largemouth bass

## Abstract

Metabolic fatty liver disease caused by high-starch diet restricted the intensive and sustainable development of carnivorous fish such as largemouth bass. In this study, the combination liver proteomic and lipidomic approach was employed to investigate the key signaling pathways and identify the critical biomarkers of fatty liver in largemouth bass. Joint analysis of the correlated differential proteins and lipids revealed nine common metabolic pathways; it was determined that FABP1 were significantly up-regulated in terms of transporting more triglycerides into the liver, while ABCA1 and VDAC1 proteins were significantly down-regulated in terms of preventing the transport of lipids and cholesterol out of the liver, leading to triglyceride accumulation in hepatocyte, eventually resulting in metabolic fatty liver disease. The results indicate that FABP1, ABCA1 and VDAC1 could be potential biomarkers for treating metabolic fatty liver disease of largemouth bass.

## 1. Introduction

Largemouth bass (*Micropterus salmoides*), originally a popular sport fish in North America, is now reared in many other countries worldwide and also is an important freshwater carnivorous species in China for its high market value as a food [1,2,3,4]. At present, more than 80% of cultured largemouth bass are fed on chilled fish [5]. However, the high costs of chilled fish as well as the potential role as disease vectors hinder the healthy development of largemouth bass aquaculture worldwide [2]. 

Previous studies discovered that fishes (especially carnivorous ones such as largemouth bass) generally have a low ability to use carbohydrate in compound feed [6,7,8]. Starch is the cheapest energy source in practical diet ingredients, and it contributes to good binders for aquatic feeds [9]. Traditional puffed floating feeds usually require at least 20% starch to obtain adequate swelling and floating properties. However, researchers demonstrated that excess dietary carbohydrate could cause fat deposition, glucose and lipid metabolism disorder, hepatopathy, apoptosis and finally result in liver damage [8,10,11,12,13]. Previous studies suggested that the starch content in the diet should be lower than 10% to ensure the liver health of largemouth bass [14,15]. Therefore, high carbohydrate is recognized as the primary factor that induced metabolic liver disease (MLD) in largemouth bass [8,9]. MLD of largemouth bass was the first limiting factor for its sustainable development worldwide [7]. Thus, it is crucial to understand the processes and progression of MLD, as well as the key factors in largemouth bass.

Proteomic technologies could be used for identifying and quantifying target proteins to comprehensively explore the etiology of liver disease, due to their high-resolution and high-throughput advantages [16]. Currently, it has become a powerful tool for understanding pathology of liver disease [17]. By identifying groups of changed proteins, we could gain insight into potential pathways and regulatory networks that might contribute to the development of MLD. Many studies have found that proteomics could be used for biomarker identification for early diagnosis in MLD [18,19,20]. To date, no study has ever comprehensively investigated the protein expression changes in fishes of MLD. Lipids are essential components for maintaining various homeostasis, physiological and cellular processes in animals [21]. Lipid metabolism disorders would lead to many major health problems, such as obesity and nonalcoholic fatty liver disease (NAFLD) [22]. Lipidomics, based on UPLC-MS/MS technology, is a novel omics strategy for investigating lipid metabolism and identifying lipid biomarkers [23,24], which enables large-scale and comprehensive studies of lipids [25]. However, there is a lack of studies that systematically assess the protein and lipid changes in the diseased liver of fish for the discovery of the biomarkers of metabolic liver disease.

In this study, we constructed a fatty liver model of largemouth bass by feeding high levels of dietary starch (16.2%) to investigate the complex biological processes and pathogenesis of MLD with proteomic and lipidomic techniques. The mass spectrometry technique was used for proteome-level quantification analyses. Some characteristic changes to different proteins and lipid metabolites are documented for the first time in this study and provide a comprehensive multi-omics framework for MLD biomarker discovery in largemouth bass. 

## 2. Materials and Methods

During the experiment period, all fishes were maintained in compliance with the Laboratory Animal Welfare Guidelines of China (Decree No. 2 of Ministry of Science and Technology, issued in 2021).

### 2.1. Experimental Diets

Two experimental diets were formulated to be isonitrogenous and isoenergetic. A basal diet was used as the control containing 10.8% starch (named the Normal group), whereas another diet was prepared with 16.2% starch (named the MLD group). Each diet was extruded into 2 mm diameter pellets using a twin-screwed extruder (EXT50A, Yang gong Machine, Beijing, China). The diet formulation and analyzed chemical composition are shown in Table 1.

### 2.2. Experimental Fish, Feeding and Sampling

Largemouth bass were obtained from the Tangshan Aquafarm (Tangshan, Hebei, China). The experiment was conducted in the indoor circulating water system at the National Aquatic Feed Safety Evaluation Base (Nan Kou, Beijing, China). Prior to the formal experiment, the fish were acclimatized for 4 weeks by being fed the Normal diet. The water temperature was maintained at 21–25 °C, pH = 7.2–8.0, dissolved oxygen (DO) >6.0 mg/L, ammonia nitrogen content <0.3 mg/L and NO_2_^−^ < 0.1 mg/L. The fish were fed until apparent satiation twice a day (8:00 am and 4:00 pm) for 10 weeks. Food intake was measured daily.

At the end of the growth trial, fishes from each group were randomly selected and anaesthetized with chlorobutanol (300 mg/mL) after 24 h starvation. The specific growth rate (SGR), final body weight (FBW) and feed conversion ratio (FCR) were detected by weighing the fish at the end of the 10 weeks. The body weight, body length, liver and viscera weight of the fish were recorded individually to calculate condition factor (CF), hepatosomatic index (HSI), viscerosomatic index (VSI) and hepatic liquid (HL), respectively. Blood samples were drawn from the caudal part of the sedated fish using anticoagulant syringes with 2% NaF and 4% potassium oxalate and centrifuged at 4000× *g* rpm for 10 min at 4 °C to obtain serum. Two parts of liver samples near to the bile duct were collected for histology examination (fixed in 4% paraformaldehyde solution) and biochemical criterion analysis (frozen in liquid nitrogen). All samples (except for histological samples) were stored at −80 °C until analysis. 

### 2.3. Hematological and Liver Homogenate Parameters

Hematological parameters included total protein (TP), total cholesterol (TC), triglyceride (TG), high-density lipoprotein cholesterol (HDL-C), low-density lipoprotein cholesterol (LDL-C), alkaline phosphatase (AKP), aspartate aminotransferase (AST), alanine aminotransferase (ALT), total bile acid (TBA) and glucose (GLU). Hepatic total antioxidative capability (T-AOC), superoxide dismutase (SOD), glutathione peroxidase (GSH-Px), catalase (CAT) and malondialdehyde (MDA) were determined by assay kits (Nanjing jiancheng Co., Nanjing, China) following the protocols given by the supplier. The reactive oxygen species (ROS) was determined by assay kit (Beyotime Biotechnology, Shanghai, China) following the protocols given by the supplier.

### 2.4. Histopathological Examination of the Liver Tissue

After 24 h of fixation, all liver samples were dehydrated by the standard procedures, and the samples were embedded in paraffin and cut to 6 μm sections. Liver sections were stained following the protocols of hematoxylin and eosin (H&E) staining and observed with light microscopy (Leica DM2500, Leica, Solms, Germany).

### 2.5. Proteomics Analysis

#### 2.5.1. Protein Extraction

The sample (3 mm × 3 mm) was grinded with liquid nitrogen into cell powder and then transferred to a 5 mL centrifuge tube. After that, four volumes of lysis buffer (8 M urea, 1% Triton-100, 10 mM dithiothreitol and 1% Protease Inhibitor Cocktail) were added to the cell powder, followed by sonication three times on ice using a high-intensity ultrasonic processor. The remaining debris was removed by centrifugation at 20,000× *g* at 4 °C for 10 min. Finally, the protein was precipitated with cold 20% TCA for 2 h at −20 °C. After centrifugation at 12,000× *g* 4 °C for 10 min, the supernatant was discarded. The remaining precipitate was washed with cold acetone three times. The protein was redissolved in 8 M urea and the protein concentration was determined with BCA kit according to the manufacturer’s instructions.

#### 2.5.2. Trypsin Digestion

For digestion, the protein solution was reduced with 5 mM dithiothreitol for 30 min at 56 °C and alkylated with 11 mM iodoacetamide for 15 min at room temperature in darkness. The protein sample was then diluted to urea concentration less than 2 M. Finally, trypsin was added at 1:50 trypsin-to-protein mass ratio for the first digestion overnight and 1:100 trypsin-to-protein mass ratio for a second 4 h digestion.

#### 2.5.3. TMT Labeling

After trypsin digestion, peptide was desalted with Strata X C18 SPE column (Phenomenex) and vacuum dried. Peptide was reconstituted in 0.5 M TEAB and processed according to the manufacturer’s protocol for TMT kit. Briefly, one unit of TMT reagent was thawed and reconstituted in acetonitrile. The peptide mixtures were then incubated for 2 h at room temperature and pooled, desalted and dried by vacuum centrifugation.

#### 2.5.4. LC-MS/MS Analysis

The hydrolysated peptides were separated by HPLC connected with a reverse capillary column. The specific steps and related conditions were as follows [19,26,27]: (1) sample desalination: 0.1% TFA cleaning for 5 min (20 μL/min); (2) gradient elution: 2–35% ACN gradient elution for 45 min (350 nL/min), 80% ACN elution for 15 min; (3) the eluent composition of HPLC: A, 0.1% formic acid solution (*v*/*v*). B, 0.1% formic acid acetonitrile solution (*v*/*v*); (4) the peptides were subjected to NSI source followed by tandem mass spectrometry (MS/MS) in Q Exactive^TM^ Plus (Thermo Fisher Scientific Co., LTD, Shanghai, China) coupled online with the UPLC. The electrospray voltage applied was 2.0 kV. The *m*/*z* scan range was 350 to 1000 for full scan, and intact peptides were detected in the Orbitrap at a resolution of 35,000. Peptides were then selected for MS/MS using NCE setting as 27 and the fragments were detected in the Orbitrap at a resolution of 17,500. A data-independent procedure alternated between one MS scan followed by 20 MS/MS scans. Automatic gain control (AGC) was set at 3E6 for full MS and 1E5 for MS/MS. The maximum IT was set at 20 ms for full MS and auto for MS/MS. The isolation window for MS/MS was set at 2.0 *m*/*z*.

#### 2.5.5. Protein Identification 

Protein identifications were performed by using the ProteinPliot software (AB Sciex). For protein quantitation, proteins were required to contain at least two unique peptides.

To demonstrate the reproducibility of the replicates, protein differential expressions between various biological replicates were compared. Then *p*-value was calculated by using the two-sample and two-tail *t*-test. When *p*-value < 0.05, the change of differential expression exceeding 1.3 was regarded as the threshold for significant up-regulation, and that less than 0.77 was regarded as the threshold for significant down-regulation.

### 2.6. Lipidomics Analysis

#### 2.6.1. Lipid Extraction

For the lipidomics analysis, the total lipids were extracted from the liver in the Normal and MLD groups. Liver tissues (20 mg) were homogenized in a 2 mL centrifuge tube with 1 mL extracting solution (methyl tertiary butyl ether: methyl alcohol = 3:1, *v*/*v*, including internal standard mixture). After homogenization, 200 μL deionized water was added and then centrifuged for 10 min at 12,000× *g* r/min, 4 °C. The supernatant solution was dried under nitrogen and redissolved in 200 μL mobile phase B for further analysis.

#### 2.6.2. UPLC-MS/MS Analysis

Lipid profiles were determined using UPLC-MS/MS (AB Sciex, Framingham, MA, USA). Samples (2 μL) were separated on Thermo Accucore^TM^C30 column (2.6 μm, 2.1 mm × 100 nm i.d.) with a flow rate of 0.35 mL/min and column temperature of 45 °C. The mobile phase consisted of a mixture of acetonitrile/water (60:40, *v*/*v*) (A) and a mixture of acetonitrile/isopropanol (10:90, *v*/*v*) (B), both containing 0.1% acetic acid and 10 mmol/L ammonium formate. The elution gradient was set stepwise as follows: 0–2 min, 20–30% B; 2–4 min, 30–60% B; 4–9 min, 60–85% B; 9–14 min, 85–90% B; 14–17.3 min, 90–95% B; 17.3–20 min, 95–20% B.

#### 2.6.3. Lipid Identification and Quantitation

The data were analyzed by multi-reaction detection mode (MRM). Conditions of MS were set as follows: ion spray voltage: +5500 V (positive) and −4500 V (negative); collision energy (CE): 35 V (positive) and −35 V (negative); temperature of electrospray ionization (ESI): 500 °C; gas 1 (GS1): 45 psi; gas 2 (GS2): 50 psi; curtain gas: 35 psi.

Fifteen standard solutions with different concentrations were prepared for quantitative analysis (concentration: 0.0002, 0.0005, 0.001, 0.002, 0.005, 0.01, 0.02, 0.05, 0.1, 0.2, 0.5, 1, 2, 5, 10 nmol/mL). Table 2 lists the lipid types and information of internal and external standards.

### 2.7. Statistical Analysis

Independent *t*-test was performed by data of growth performance. All data are presented as the mean value ± standard error of the mean (S.E.M). *p* < 0.05 was considered significantly different. The correlation and linear regression analysis of biological repetitions were carried out using GraphPad Prism 8.0 (Graph- Pad Software Inc., San Diego, CA, USA).

Functional annotations of the proteins and genetic ontology were conducted using the Blast2GO program. The related pathways of each protein and bioinformation mining were analyzed by searching the KEGG pathway database.

R software was used for Principal Component Analysis (PCA), cluster analysis, grouping Principal Component Analysis, differential metabolite screening and KEGG functional annotation of different samples.

## 3. Results

### 3.1. High-Starch Diet (HSD) Induced Fatty Liver of Largemouth

**Figure 1:**
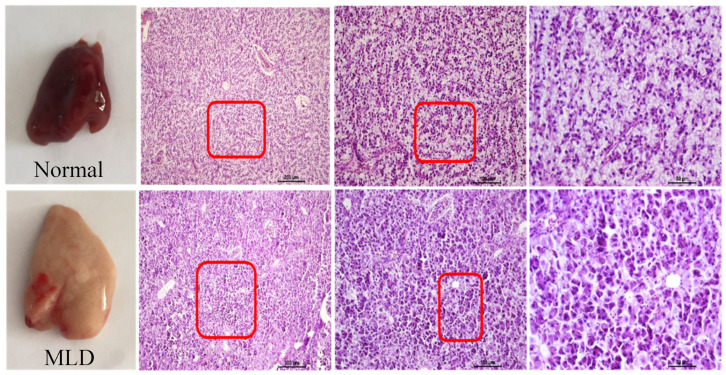
Liver histopathology, enlarged lipid droplets (marked with red boxes) were clearly observed in MLD group. (MLD: metabolic liver disease).

Fish liver sections were examined after H&E staining and Sirius red staining for collagen. Typical phenotypes of normal liver and fatty liver are shown in Figure 1. The results showed that HSD led to enlarged lipid droplets and vacuolated cells.

Both Normal and MLD groups showed high survival (99%) and there was no significant difference between the two groups. Compared with the Normal group, fish in the MLD group showed significantly higher HSI and HL (*p* < 0.05) but significantly lower SGR and FBW (*p* < 0.05, Table 3).

LDL-C indicators (in plasma and liver, Table 4 and Table 5) and the hepatic function indicators (AKP, AST and ALT) increased significantly in the MLD group (*p* < 0.05, Table 4). Higher ROS level and lower CAT content were recorded in the MLD group (Table 6).

### 3.2. Proteomics Analysis

**Figure 2:**
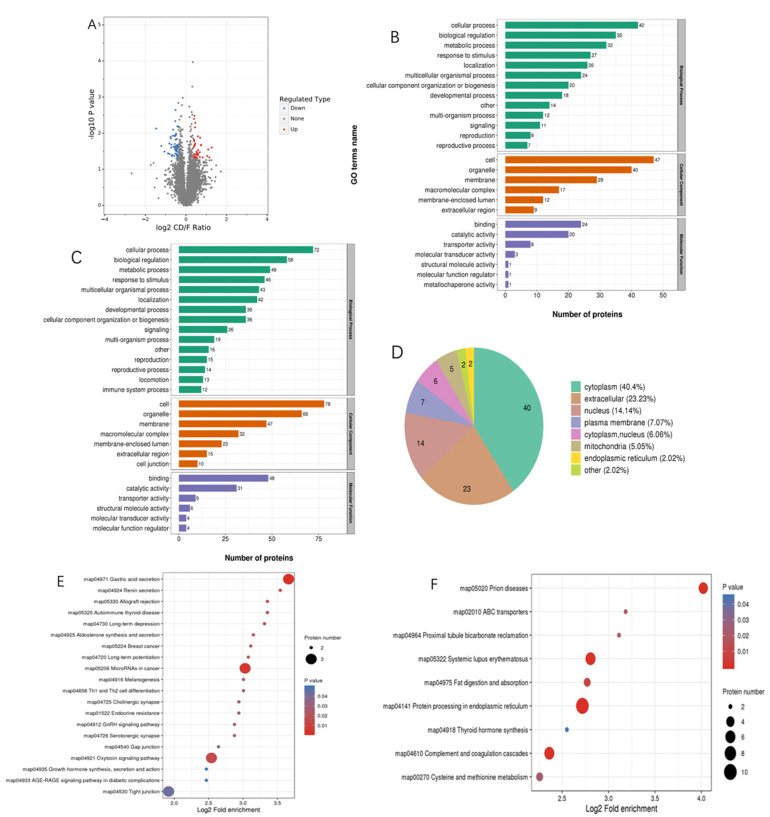
Significantly different expressions between MLD and Normal groups. (**A**) Protein significantly different expression between MLD and Normal groups (red means up-regulated, blue means down-regulated). (**B**,**C**) Differentially expressed proteins in GO functional classification (**B**: up; **C**: down). (**D**) The subcellular localization of differentially expressed proteins. (**E**,**F**) KEGG enrichment pathways of different expressed proteins (**E**: up-regulated; **F**: down-regulated).

Liver proteome profiles of largemouth bass with fatty liver (*n* = 8) and normal liver were compared (*n* = 8) (Table S1). This revealed an obvious shift in the proteome composition, reflected by 99 significantly differentially abundant proteins (Fold change >1.3 or <0.77), of which 38 proteins were up-regulated and 61 down-regulated (Figure 2A).

To investigate the functions of the identified and quantified proteins, we annotated their functions and features, gene ontology (GO), subcellular localization, GO-based enrichment, Kyoto Encyclopedia of Genes and Genomes (KEGG) pathway analysis, protein domain and involvement in a protein complex of the characteristics we annotated [26]. 

For the biological process category, the differential proteins between the Normal and MLD groups were related to cellular processes, biological regulation processes, metabolic processes, response to stimulus processes, multicellular organismal processes, localization processes and development processes. For the cellular component category, these differential proteins were mainly enriched in the organelle, membrane, macromolecular complex and membrane-enclosed lumen. For the molecular function category, fatty liver induced by high-starch diet was primarily enriched in functional clusters, such as catalytic activity, transporter activity and structural molecule activity. (Figure 2B,C).

Subcellular structure localizations of differentially expressed proteins were predicted and classified, the results showed that most of these differential proteins were located in the nucleus, cell membrane, cytoplasmic cells and external matrix, and a few were located in mitochondria and endoplasmic reticulum. Forty were located in the cytoplasm of the 99 differential expressed proteins, with 23 in the extracellular matrix, 14 in the nucleus, 7 in the cell membrane, 6 in the cytoplasm and nucleus, 5 in the mitochondria, 2 in the endoplasmic reticulum and 2 in other cellular structures (Figure 2D).

For the KEGG pathway analysis, differential proteins were primarily associated with enrichment in pathways related to the fat digestion and absorption, gastric acid secretion, ABC transport, cholesterol metabolism, glycolysis/gluconeogenesis, PPAR and MAPK (Figure 2E,F). The differential pathways that may be related to fatty liver metabolism and the important differentially expressed proteins in these pathways are listed in Table 7.

### 3.3. Lipidomics Analysis

**Figure 3:**
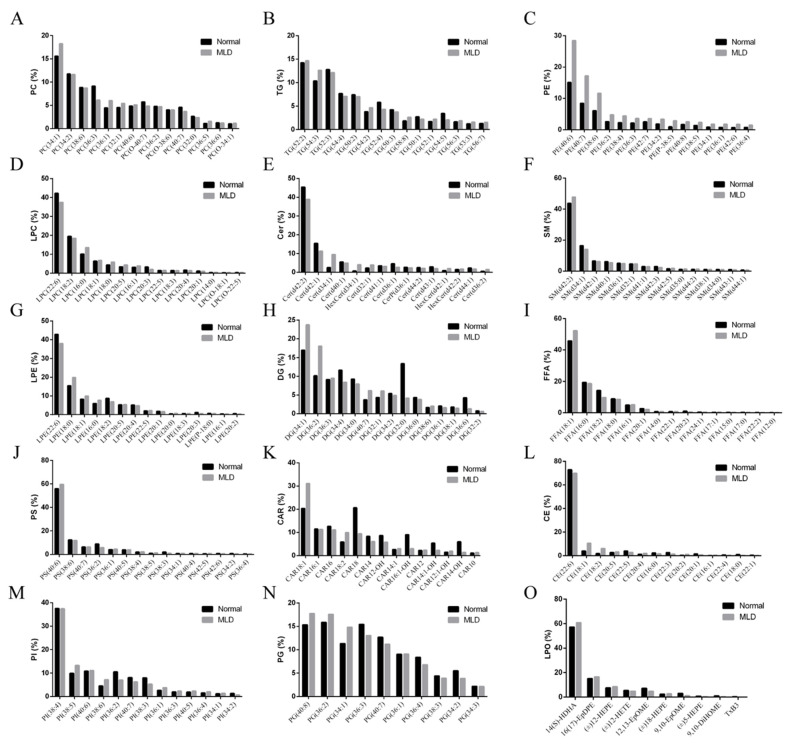
Major lipid in the liver of largemouth bass (Mean ± SEM, *n* = 8). (**A**) PC: phosphatidylcholine. (**B**) TG: triacylglycerol. (**C**) PE: phosphatidylethanolamine. (**D**) LPC: lysophosphatidyl choline. (**E**) Cer: ceramide. (**F**) SM: sphingomyelin. (**G**) LPE: lysophosphatidylethanolamine. (**H**) DG: diacylglycerol. (**I**) FFA: free fatty acid. (**J**) PS: phosphatidylserine. (**K**) CAR: acyl carnitine. (**L**) CE: cholesteryl ester. (**M**) PI: phosphatidylinositol. (**N**) PG: phosphatidyl glycerol. (**O**) LPO: lipid peroxidation.

For liver lipid analysis, 15 lipid species including phosphatidylcholine (PC), triacylglycerol (TG), phosphatidylethanolamine (DPPE), lysophosphatidyl choline (LPC), ceramide (Cer), sphingomyelin (SM), lysophosphatidylethanolamine (LPE), diacylglycerol (DG), free fatty acid (FFA), phosphatidylserine (PS), acyl carnitine (CAR), cholesteryl ester (CE), phosphatidylinositol (PI), phosphatidyl glycerol (PG) and lipid peroxidn (LPO) were detected with lipidomics analysis (74 PCs, 69 TGs, 55 DPPEs, 37 LPCs, 36 Cers, 32 SMs, 21 LPEs, 17 DGs, 16 FFAs, 15 PSs, 14 CARs, 14 CEs, 13 PIs, 10 PGs, 10 LPOs). The major lipid compositions were analyzed (Table 8 and Figure 3).

**Figure 4:**
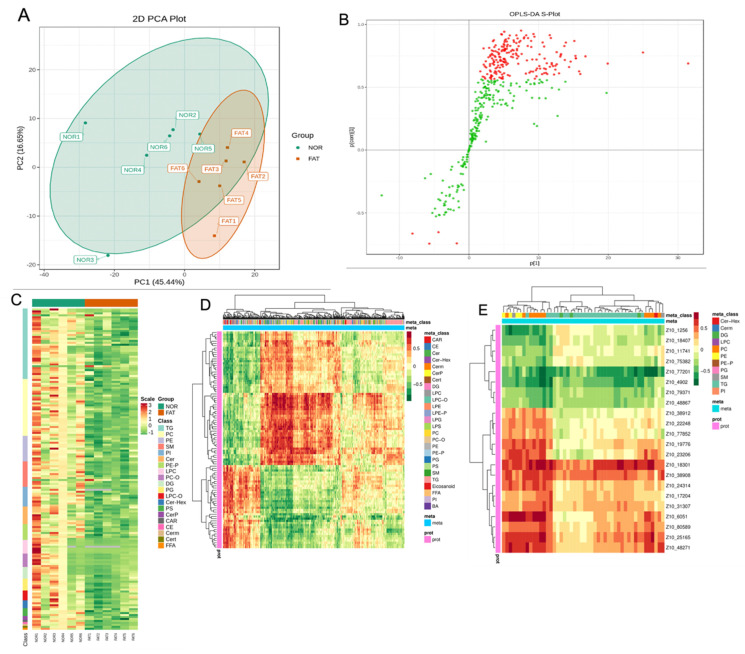
The lipidomics analysis of largemouth bass liver. (**A**) Principal component analysis (PCA) score plots. (**B**) OPLS-DA analysis (red points and green points represent VIP ≥ 1 and VIP ≤ 1, respectively) and (**C**) heatmap. (Mean ± SEM, *n* = 8). (**D**) Heatmap of all lipids and proteins (red represents high correlation coefficient; green represents low correlation coefficient). (**E**) Heatmap of correlation coefficient of differential lipids and differential proteins (red represents a positive correlation between proteins and lipids and green represents a negative correlation). Each row and column represents a protein and a metabolite, respectively.

PCA analysis showed discrimination between the MLD group and the Normal group (Figure 4A), indicating that there was difference in the lipid composition between the two groups. OPLS-DA analysis was applied to screen out the differential metabolites between the two groups. Using the criteria of VIP ≥ 1 and FC ≥ 1.5 or ≤ 0.67, 164 significantly differential lipid species were identified in the Normal versus the MLD group (Figure 4B). As shown in the heatmap (Figure 4C), the differential lipid species were mainly enriched in TC, PC, DPPE, Cer, SM, PI and DG classes, and all of them were upregulated in the MLD group. Notably, the top 20 differential upregulated lipids were mainly TGs, including TG (51:0), TG (51:1), TG (54:1), TG (50:0), TG (52:0), TG (49:1) and TG (54:0). However, the three downregulated lipids were mainly LPC (20:2), Cer (d34:1) and PE (P-34:2) (Table 9).

A total of 29 differential metabolic pathways were found with KEGG pathway enrichment analysis. These metabolic pathways mainly involved fat digestion and absorption, cholesterol metabolism, vitamin digestion and absorption, sphingolipid metabolism and insulin resistance, all of which are related to lipid metabolism (Table 10).

### 3.4. Integrative Proteomic and Lipidomic Analysis

To best observe the correlation between lipid metabolites and proteins, all the lipids and proteins were analyzed by clustering analysis as illustrated in a heatmap (Figure 4D). The result indicated that there are correlations between many lipids and proteins. All the differential proteins and differential lipids were selected, and the heatmap clustering was drawn. As shown in Figure 4E, these differential proteins were negatively correlated with differential lipids, including aldo-keto reductase family 1 member D1 (AK1R1D1), phosphoenolpyruvate carboxykinase (PEPCK), ATP binding cassette transporter A1 (ABCA1), beta-1,3-N-acetylglucosaminyltransferase 3 (B3GNT3), L-2-hydroxyglutarate dehydrogenase (L2HGDH), cytochrome P450 2U1 (CYP2U1), mercaptopyruvate transferase, phosphatidylinositol 4-kinase beta (PI4K2β), hexose transferase, cystathionine beta-synthase (CBS) and voltage-dependent anion channels1 (VDAC1). These differential proteins were positively correlated with differential lipids, namely hexose tetraphosphate kinase, natriuretic peptide receptor-A (NPR-A), fatty acid binding protein 1 (FABP1), UDP-glucuronosyltransferase (UGT), fatty acid acetyl-CoA synthase (FA-CoA) and Guanine nucleotide-binding protein G(q) subunit alpha (GNAQ). Among these differential lipid metabolites, we observed that the strongest relationships with differential proteins were triglyceride (TG) and phosphatidylcholine (PC).

The above correlated differential lipids and differential proteins were simultaneously mapped to the KEGG pathway, and were enriched into nine common metabolic pathways, as shown in Table 11. Four KEGG pathways with the highest concentration were selected for subsequent analysis, including thermogenesis (ko04714), fat digestion and absorption (ko04975), cholesterol metabolism (ko04979) and metabolic pathways (ko01100). 

## 4. Discussion

Carnivorous fish have a low starch utilization rate, and high digestible starch intake leads to accumulation of liver glycogen and persistent high blood sugar, finally causing fish liver disease [28,29]. Generally, the level of digestible carbohydrate above 10% reduced the growth performance of largemouth [9,15,30,31], induced glycogen and lipid accumulation and dysfunction of antioxidant capabilities, leading to MLD in largemouth [9,32]. In this study, compared with the Normal group (starch content: 10.8%), the MLD group (starch content: 16.2%) decreased the growth performance of largemouth bass and induced liver lipid accumulation, liver function injury, oxidative stress and higher hepatosomatic index, which is similar to the non-alcoholic fatty liver symptom. Therefore, we used HSD to construct the fatty liver phenotype in largemouth bass successfully. 

### 4.1. Proteomic Analysis

The differentially expressed proteins induced by a high-starch diet of largemouth bass were mainly involved in endoplasmic reticulum protein processing, fat digestion and absorption, cholesterol metabolism, phosphatidylinositol signal system and insulin signal transduction, which affected the occurrence and development of fatty liver through the comprehensive action of multiple metabolic pathways. 

Endoplasmic reticulum (ER) is an important site involved in the regulation of substance transport, metabolism and protein synthesis in eukaryotic cells [33]; ER stress caused by various reasons is closely related to the occurrence and development of diseases [34]. The evidence implied that ER played a role in the development of steatosis and nonalcoholic steatohepatitis. ER stress occurred in liver and adipose tissue in patients with non-alcoholic fatty liver disease [35,36]. RRBP1 (reticulum ribosome-binding protein 1) is one of the important proteins involved in the ER unfolded protein reaction, and abnormal up-regulated expression of RRBP1 has been found in the study of human cancer-related diseases [37,38,39]. Xiong found that the expression of RRBP1 in liver cancer tissues was significantly higher than that in normal liver tissues, and the expression of RRBP1 in liver cancer tissues with metastasis was significantly higher than that in liver cancer tissues without metastasis, indicating that RRBP1 is closely related to liver cancer metastasis in the state of liver injury [40]. In this study, RRBP1 was found to be highly expressed in liver in the MLD group, indicating that it may have an important relationship with liver injury and the development of MLD. In addition, TRAPα (translocon-associated protein subunit alpha) is a typical glycosylated membrane protein which plays an important role in the process of signal modification recognition and the transport of new peptide chains. It synergizes with AMPK (adenosine monophosphate activated protein kinase) and ROS pathway to cause resistance to oxidative stress-induced apoptosis [41]. TRAPα also plays an important role in maintaining cell homeostasis and may become a target drug for intervention in related diseases [42]. The low expression of TRAPα may be due to the abnormality in the modification and transport of the peptide chain, resulting in liver damage, which affects the normal progress of subsequent life activities and leads to the occurrence and development of fatty liver. At present, the TRAPα sequence of fish is relatively conserved, but the research on its related functions is still in the blank stage and needs to be further studied.

The pathway of fat digestion and absorption has the important function of maintaining the homeostasis of lipid metabolism. In this study, we found the differential expression of three important proteins FABP1, MTTP (microsomal triglyceride transfer protein) and ABCA1 in its pathway. FABP1 is expressed mainly in the liver and could combine with long-chain fatty acid to regulate lipid absorption and fatty acid metabolism in the cytoplasm [43]. FABP1 activity changed significantly in the occurrence and development of fatty liver, liver cirrhosis, liver cancer and other liver diseases [44,45,46]. In this study, HSD induced fatty liver of largemouth bass, leading to significant upregulation of FABP1. Similar results also showed that FABP1 levels were higher in patients with non-alcoholic fatty liver disease [43,47]. Therefore, FABP1 is expected to become a diagnostic marker of liver injury [48,49,50]. The MTTP is a key protein for lipid excretion in liver and affects the metabolism of lipids and lipoproteins, leading to the occurrence and development of many diseases [51,52,53,54]. Specific knockout of the MTTP gene in hepatocytes leads to the accumulation of a large amount lipids in mouse hepatocytes, and eventually results in hepatic steatosis and fatty liver [55]. Some evidence showed that the variability of MTTP is associated with the development of NAFLD, raised lipid and risk of atherosclerotic cardiovascular disease [56,57]. A previous study indicated that in the NAFLD of rat induced by a high-fat diet, the expression level of MTTP is markedly decreased [58]. Similarly, HSD-induced fatty liver of largemouth bass decreased MTTP level in liver in our results. ABCA1 played an essential role in the regulation of high-density lipoproteins (HDLs) and reverse cholesterol transport [59]. ABCA1 overexpression increased the concentration of cholesterol and high-density lipoprotein and decreased hepatic lipid contents [60,61]. The deposition of cholesterol in tissues induced the absence of ABCA1 [62,63]. A previous study reported that decreased hepatic ABCA1 expression can cause steatohepatitis in obese adult patients [61]. It also presented the evidence that the expression level of ABCA1 was significantly lower in the NAFLD patients than in the healthy controls [64]. Consistent with these observations, ABCA1 was down-regulated in the MLD group induced by HSD. These findings suggest that the expression level of ABCA1 was associated with the development of fatty liver disease. In this study, the differential expression of FABP1, ABCA1 and MTTP in the liver of largemouth bass in the MLD group showed a high-starch diet increased hepatic lipid contents and affected fat digestion and absorption pathways, and eventually promoted the development of metabolic fatty liver. 

### 4.2. Lipidomic Analysis

Significant changes in hepatic lipids are important pathophysiological markers of fatty liver [65]. Lipidomic studies of the mouse liver have shown that NAFLD is associated with an increase in TG [66]. Similar results were observed in our HSD treatment largemouth bass. In this study, most TG species were increased by HSD. During the occurrence of fatty liver, the up-regulation of TGs is attributed to the rate of TG synthesis in the liver exceeding the catabolic rate of TGs [67,68]. Therefore, preventing TG accumulation in liver may contribute to the attenuating effect of fatty liver disease [66,69].

KEGG pathway enrichment analysis indicated that the insulin-resistance pathway was significantly enriched. Many studies believe that NAFLD is closely related to insulin resistance [70,71,72]. The relationship between NAFLD and insulin resistance is bidirectional. Insulin resistance may promote the development of NAFLD, while NAFLD may promote the development of insulin resistance [71,73]. The related research results of our team also found that the persistent hyperglycemia of largemouth bass after a meal is caused by insufficient insulin secretion (unpublished), which is also the reason for the starch intolerance of largemouth bass. Therefore, insulin resistance is a key factor in the development and progression of NAFLD [74] and may be the direction of targeted intervention of MLD in largemouth bass in the future. 

Interestingly, in this study we also found that differential lipids were mainly enriched in the sphingolipid metabolism signaling pathway. Liver is the center of ceramide production and usually contains much higher sphingolipid than all other tissues, especially ceramide and sphingomyelin [75,76,77]. Therefore, liver is prone to sphingolipid toxicity [78]. For example, sphingolipid content was significantly increased in the liver of rats fed with a high-fat diet [79]. Mice exposure to exogenous microbiome sphingolipids no longer had lipid accumulation and rescued diet-induced hepatic steatosis [80]. Evidence suggested sphingolipid levels contribute to the development of NAFLD in a variety of ways, including inflammation, insulin resistance and oxidative stress [78].

### 4.3. Integrative Proteomic and Lipidomic Analysis

In this study, using integrative proteomic and lipidomic analysis, we demonstrated the hepatic TG accumulation was important for the development progress of fatty liver induced by HSD. Theoretically, the primary cause of lipid accumulation is the increased uptake of free fatty acids (FFAs) by the liver [56,81]. Undoubtedly, regulation of TG accumulation is important for improving fatty liver disease [82]. Many studies have shown that overexpression of FABP1 enhances fatty acid uptake [83,84,85]. Researchers also found FABP1-deficient mice were protected from TG accumulation in liver induced by a high-fat diet [86,87]. It is expected that reducing the expression or function of FABP1 could inhibit TG accumulation in liver and ameliorate NAFLD [85]. In this study, we similarly observed HSD increased the expression level of FABP1, resulting in more TG accumulation in liver, ultimately leading to fatty liver in largemouth bass.

Meanwhile, accumulated evidence suggested cholesterol homeostasis and hepatic cholesterol accumulation associated with the risk of liver cirrhosis and other liver diseases [88,89,90]. ABCA1 could prevent cholesterol accumulation by transporting cholesterol and phospholipids from cells to apolipoproteins [91,92,93]. Recently, a report showed that ABCA1 were tightly associated with the lipid variables and lipid metabolism [94]. Human studies also suggest the elevation of TG levels usually develop fatty liver disease, and there is an inverse association between TG concentrations and dysfunctional ABCA1 [95]. In addition, a study also reported when the function of ABCA1 is diminished, the TG secretion from liver increased, suggesting there is a strong association between ABCA1 and fatty liver disease [59]. Consistent with this finding, this study also showed the content of ABCA1 was significantly reduced, which exacerbated the accumulation of cholesterol in the liver and worsened metabolic fatty liver disease.

Furthermore, VDAC1 was involved in cholesterol transport and was thought to be part of a complex process that mediates fatty acid transport [96,97,98]. A lack of VDAC1 caused mitochondria to stop oxidizing fatty acids [99]. It is also indicated that the absence of ABCA1 lead to steatosis in metabolic liver disease [100,101]. Recent study had found hepatocyte fat droplet and glycogen accumulation in high-fat diet-treated mice. However, the VDAC1-based peptide decreased serum TG content and hepatocyte fat droplets and increased hepatocyte glycogen accumulation [98]. Moreover, VDAC1 could interact with many proteins, such as metabolism, apoptosis and anti-oxidation-related proteins [102,103], so it could be regarded as a potential therapeutic for metabolic liver disease, such as NAFLD [98]. Here, we demonstrated that the reduction in VDAC1 expression led to cholesterol and lipid accumulation in liver of largemouth bass induced by HSD. Importantly, in addition to hepatic TG accumulation, abnormal transport of cholesterol also plays a critical role in the occurrence and development of fatty liver disease. 

## 5. Conclusions

We successfully induced metabolic fatty liver phenotype of largemouth bass with high-starch diet. We found that three differential proteins (FABP1, ABCA1 and VDAC1) in fat digestion and absorption and cholesterol metabolism pathways played important roles in the occurrence and development of MLD in largemouth bass after the combination of targeted lipidomics and proteomics analyses. The three differential proteins may be promising targets for therapeutic intervention in fatty liver disease. Our findings propose the possible key regulatory factors in the occurrence and development of metabolic fatty liver, which provide a theoretical basis for the efficient use of compound feed and targeted nutritional regulation in commercial aquaculture.

## Figures and Tables

**Table 1:** Formulation and composition of experimental diets (%).

Ingredients (in Dry Matter Basis, %)	Normal Group	MLD Group
^a^ Fish meal	30.0	30.0
Tapioca starch	5	5
Wheat flour	9.0	18
Microbial protein	4	4
Cottonseed concentrate protein	23.5	22.6
Wheat gluten meal	4	4
Soybean meal	2	-
Spay-dried blood cell powder	4	4
α-cellulose	4.6	-
Ca(H_2_PO4)_2_	1.7	1.7
Lecithin oil	2.0	2.0
Fish oil	3.5	3.5
Soybean oil	3.5	3.5
Kelp powder	1.5	0
*L*-Threonine	0.1	0.1
*DL*-Methionine	0.2	0.2
^b^ Vitamin and mineral premix	1.4	1.4
Total	100	100
Nutrients compositions		
Crude protein	50.83	51.15
Ether extract	12.36	12.33
Crude ash	10.08	10.04
Moisture	6.10	7.43
Gross energy (MJ/Kg)	20.45	20.15

^a^ Fish meal: crude protein content was 68.8%; soybean protein concentrate: crude protein content was 65.2%; cottonseed protein concentrate: crude protein content was 61.5%. ^b^ Vitamin and mineral premix (mg/kg diets): VA 20 mg, VK_3_ 20 mg, VD_3_ 10 mg, VE 400 mg, VB_1_ 10 mg, VB_2_ 15 mg, VB_6_ 15 mg, VB_12_ (1%) 8 mg, VC (35%) 1000 mg, Calcium pantothenate 40 mg, Inositol 200 mg, Niacinamide 100 mg, Biotin (2%) 2 mg, Folic acid 10 mg, Corn gluten meal 150 mg; Choline chloride (50%) 4000 mg; FeSO_4_·H_2_O 300 mg, ZnSO_4_·H_2_O 200 mg, MnSO_4_·H_2_O 100 mg, CoCl_2_·6H_2_O (10%Co) 5 mg, KI (10%) 80 mg, Na_2_SeO_3_ (10% Se) 10 mg, MgSO_4_·5H_2_O 2000 mg, Zeolite 4995 mg, NaCl 100 mg, Antioxidant 200 mg, CuSO_4_·5H_2_O 10 mg.

**Table 2:** Lipid types and information of part of internal and external standards.

	Lipids	Abbreviation	CAS	Standards Type
1	Lysophosphatidyl choline	LPC (13:0)	20559-17-5	Internal
2	Cholesterol heptadecanoate	CE (17:0)	24365-37-5	Internal
3	Ceramide C4	Cer (d18:1/4:0)	74713-58-9	Internal
4	Diester of glycerol dodecanoate	DG (12:0/12:0)	60562-15-4	Internal
5	Lysophosphatidyl ethanolamine	LPE (14:0)	123060-40-2	Internal
6	Phosphatidylcholine	PC (13:0/13:0)	71242-28-9	Internal
7	Phosphatidyl ethanolamine	PE (12:0/12:0)	59752-57-7	Internal
8	Diphosphatidyl glycerol	PG (12:0/12:0)	322647-27-8	Internal
9	Phosphatidylserin	PS (14:0/14:0)	105405-50-3	Internal
10	Triglyceride dodecyl	TG (12:0/12:0/12:0)	555-44-2	Internal
11	Phosphatidyl inositol	PI (16:0/16:0)	34290-57-8	Internal
12	Palmitic acid -d31	FFA (16:0)-d31	39756-30-4	Internal
13	Cholesterol linoleate	CE (18:2)	604-33-1	External
14	Ceramide C17	Cer (d18:1/17:0)	67492-16-4	External
15	Diester of glycerol hexadecanoate	DG (16:0/16:0)	30334-71-5	External
16	Lysophosphatidyl choline	LPC (17:0)	50930-23-9	External
17	Lysophosphatidyl ethanolamine	LPE (16:0)	53862-35-4	External
18	Phosphatidyl choline	PC (17:0/17:0)	70897-27-7	External
19	Phosphatidyl ethanolamine	PE (17:0/17:0)	140219-78-9	External
20	Phosphatidyl glycerol	PG (17:0/17:0)	799268-52-3	External
21	Phosphatidylserine	PS (17:0/17:0)	799268-51-2	External
22	Sphingomyelin	SM (d18:1/17:0)	121999-64-2	External
23	Triglyceride heptadecanoate	TG (17:0/17:0/17:0)	2438-40-6	External
24	Phosphatidyl inositol	PI (16:0/18:1)	50730-13-7	External
25	Palmitic acid	FFA (16:0)	57-10-3	External

**Table 3:** Changes of MLD on morphometric parameters of largemouth bass (means ± SEM).

Items	Normal	MLD
CF (g/cm^3^)	2.03 ± 0.09	2.01 ± 0.08
VSI (%)	7.17 ± 1.86	7.36 ± 0.20
HSI (%)	1.66 ± 0.08 ^b^	2.36 ± 1.17 ^a^
FBW (g)	105.83 ± 1.68 ^b^	95.54 ± 1.30 ^a^
SGR	2.00 ± 0.31 ^b^	1.80 ± 0.03 ^a^
FCR	0.98 ± 0.01	1.00 ± 0.18
HL	6.54 ± 0.29 ^b^	7.39 ± 0.10 ^a^

^a, b^ Within the same column, values with different superscripts are significantly different (*p* < 0.05). The same as below, *n* = 8; CF (condition factor, g/cm^3^) = 100 × average body weight/average body length^3^; VSI (viscerosomatic index, %) = 100 × visceral weight/whole body weight; HSI (hepatosomatic index, %) = 100 × liver weight/whole body weight; FBW: final body weight, *n* = 8; SGR (specific growth rate, %) = 100 × [ln (FBW/initial body weight)]/days, *n* = 8; FCR (feed conversion rate) = FI_abs/_[(final total weight − initial total weight)/days]; Where, FI_abs_ is the daily absolute feed intake; HL (hepatic liquid, %) = fat weight of liver/liver weight.

**Table 4:** Changes of MLD on plasma liver function parameters of largemouth bass (means ± SEM).

Items	Normal	MLD
TP (g/L)	16.49 ± 0.47	15.69 ± 0.50
GLU (mmol/L)	5.71 ± 0.35 ^b^	4.25 ± 0.45 ^a^
TG (mmol/L)	5.64 ± 1.18	5.48 ± 0.45
TC (mmol/L)	7.70 ± 0.79	8.16 ± 0.62
HDL-C (mmol/L)	1.75 ± 0.38	1.64 ± 0.32
LDL-C (mmol/L)	2.00 ± 0.20 ^b^	2.24 ± 0.13 ^a^
AKP (U/L)	49.10 ± 5.56 ^b^	77.34 ± 5.13 ^a^
AST (U/L)	5.87 ± 1.05 ^b^	11.15 ± 1.88 ^a^
ALT (U/L)	5.87 ± 1.04 ^b^	12.65 ± 1.97 ^a^
TBA (umol/L)	74.41 ± 1.12 ^b^	78.11 ± 4.49 ^a^

^a, b^ Within the same column, values with different superscripts are significantly different (*p* < 0.05). The same as below; TP, total protein; GLU, glucose; TG, triglyceride; TC, total cholesterol; HDL-C, high-density lipoprotein cholesterol; LDL-C, low-density lipoprotein cholesterol; AKP, alkaline phosphatase; AST, aspartate aminotransferase; ALT, aminotransferase; TBA, total bile acid. Within the same row, values with different superscripts are significantly different.

**Table 5:** Effects of MLD on hepatic lipid metabolism of largemouth bass (means ± SEM).

Items	Normal	MLD
TG (mmol/g·prot)	0.17 ± 0.01 ^b^	0.21 ± 0.05 ^a^
TC (mmol/g·prot)	0.15 ± 0.02	0.15 ± 0.01
TBA (umol/mg·prot)	2.33 ± 0.28	2.33 ± 0.65
LDL-C (umol/g·prot)	30.03 ± 2.92 ^b^	43.92 ± 4.89 ^a^
LDL-C/TC	0.22 ± 0.03	0.29 ± 0.03

^a, b^ Within the same column, values with different superscripts are significantly different (*p* < 0.05).

**Table 6:** Effects of MLD on hepatic antioxidant responses of largemouth bass (means ± SEM).

Items	Normal	MLD
ROS (U/mg·prot)	66.78 ± 4.92 ^b^	88.14 ± 4.85 ^a^
T-AOC (umol/g·prot)	76.78 ± 5.96	89.14 ± 9.02
CAT (U/mg·prot)	46.62 ± 2.07 ^a^	31.13 ± 3.42 ^b^
GSH-Px (U/ug·prot)	4.07 ± 0.51	4.38 ± 0.57
SOD (U/mg·prot)	182.40 ± 9.45	168.75 ± 19.81
MDA (nmol/mg·prot)	2.69 ± 0.71	3.00 ± 0.38

^a, b^ Within the same column, values with different superscripts are significantly different (*p* < 0.05).

**Table 7:** Selected KEGG pathway analysis of differentially expressed proteins related to metabolic fatty liver.

KEGG Pathway	Related Proteins (Up-Regulated)	Related Proteins (Down-Regulated)
Protein processing in endoplasmic reticulum	RRBP1	TRAPα, PDIA4
Fat digestion and absorption	FABP1	ABCA1, MTTP
ABC transporters		ABCA1
Cholesterol metabolism		ABCA1, VDAC1, AK1R1D1
PPAR signal pathway	HRAs, FABP1	PEPCK
FoxO signaling pathway;	HRAS	PEPCK
mTOR signaling pathway	HRAs	
Glycolysis/Gluconeogenesis		PEPCK
Phosphatidylinositol signalingsystem		PI4Kβ
Metabolic pathways	FA-CoA, UGT, lipocalin	PEPCK, CBS, L2HGDH, B3GNT3, PI4Kβ, CYP2U1, AK1R1D1
Insulin signaling pathway	HRAs, GNAQ	PEPCK
Thyroid hormone synthesis	GNAQ	PDIA4
Primary bile acids synthesis		AK1R1D1
Themogenesis	HRAs	NPR-A

**Table 8:** Major lipid composition in the liver of largemouth bass.

Classification	Subclass	Composition
Fatty acyl	Free fatty acid	FFA(18:1), FFA(16:0), FFA(18:2), FFA(18:0), FFA(16:1), FFA(20:1), FFA(14:0), FFA(22:1), FFA(20:2), FFA(24:1)
	Acyl carnitine	CAR18:1, CAR16:1, CAR16, CAR18:2, CAR18, CAR14, CAR12-OH, CAR14:1, CAR18:1-OH, CAR12
Glyveride	Diacylglycerol	DG(34:1), DG(36:2), DG(36:3), DG(34:4), DG(34:0), DG(40:7), DG(32:1), DG(34:2), DG(32:0), DG(36:0)
	Triacylglycerol	TG(52:2), TG(54:3), TG(52:3), TG(54:5), TG(50:2), TG(54:2), TG(52:4), TG(50:3), TG(58:8), TG(50:1)
Glyceryl phosphatide	Lysophosphatidyl choline	LPC(22:6), LPC(18:2), LPC(16:0), LPC(18:1), LPC(18:0), LPC(20:5), LPC(16:1), LPC(20:3), LPC(22:5), LPC(18:3)
	Lysophosphatidyl ethanolamine	PE(40:6), PE(40:7), PE(38:6), PE(36:2), PE(38:4), PE(36:3), PE(42:7), PE(34:2), PE(P-38:5), PE(40:8), LPE(22:6), LPE(18:0), LPE(18:1), LPE(16:0), LPE(18:2), LPE(20:5), LPE(20:4), LPE(22:5), LPE(20:1), LPE(20:0)
	Phosphatidyl choline	PC(34:1), PC(34:2), PC(38:6), PC(36:3), PC(36:1), PC(32:1), PC(40:6), PC(O-40:7), PC(36:2), PC(O-38:6)
	Phosphatidyl glycerol	PG(40:8), PG(36:2), PG(34:1), PG(36:3), PG(40:7), PG(36:1), PG(36:4), PG(38:3), PG(34:2), PG(34:3)
	Phosphatidylinositol	PI(38:4), PI(38:5), PI(40:6), PI(38:6), PI(36:2), PI(40:7), PI(38:3), PI(36:1), PI(36:3), PI(40:5)
	Phosphatidylserine	PS(40:6), PS(38:6), PS(40:7), PS(36:2), PS(36:1), PS(40:5), PS(38:4), PS(38:5), PS(38:3), PS(34:1)
Sphingolipid	Sphingomyelin	SM(d42:2), SM(d34:1), SM(d42:1), SM(d40:1), SM(d36:1), SM(d32:1), SM(d41:1), SM(d42:3), SM(d42:5), SM(d35:0)
	Ceramide	Cer(d42:2), Cer(d42:1), Cer(d34:1), Cer(d40:1), HerCer(d34:1), Cer(d32:1), Cer(d41:1), Cer(d36:1), CerP(d36:1), Cer(d44:2)
Cholesterol	Cholesteryl ester	CE(22:6), CE(18:0), CE(18:1), CE(18:2), CE(20:5), CE(22:5), CE(20:4), CE(16:0), CE(22:3), CE(20:2), CE(20:1)

**Table 9:** Top 20 differential up-regulated and down-regulated metabolites.

Metabolites	Class	VIP	*p*-Value	Log2FC	Type
TG (51:0)	TGs	1.22	N/A	4.50	up
TG (51:1)	TGs	1.22	0.09	3.44	up
TG (54:1)	TGs	1.11	0.09	2.93	up
TG (50:0)	TGs	1.25	0.07	2.79	up
TG (52:0)	TGs	1.19	0.13	2.64	up
TG (49:1)	TGs	1.29	0.03	2.62	up
TG (54:0)	TGs	1.09	0.16	2.53	up
TG (52:1)	TGs	1.17	0.06	2.49	up
TG (48:0)	TGs	1.32	0.03	2.43	up
TG (46:0)	TGs	1.34	0.02	2.39	up
TG (56:0)	TGs	1.09	0.12	2.37	up
TG (52:7)	TGs	1.52	0.02	2.33	up
TG (53:2)	TGs	1.07	0.06	2.26	up
TG (58:7)	TGs	1.32	0.04	2.21	up
TG (44:0)	TGs	1.18	0.03	2.14	up
TG (56:9)	TGs	1.21	0.07	2.02	up
TG (58:10)	TGs	1.14	0.05	1.92	up
DG (38:6)	DGs	1.33	0.00	2.03	up
PI (38:5)	PIs	1.54	0.00	2.25	up
PE (P-40:5)	PEs	1.47	0.02	2.37	up
LPC (20:2)	LPCs	1.32	0.03	0.96	down
Cer (d34:1)	Cers	1.16	0.05	0.72	down
PE (P-34:2)	PEs	1.17	0.05	1.38	down

**Table 10:** Differential metabolic pathways.

Pathways	ko_ID	Unique Compound
Metabolic pathways	ko01100	99
Insulin resistance	ko04931	36
Sphingolipid signaling pathway	ko04071	21
Fat digestion and absorption	ko04975	29
Cholesterol metabolism	ko04979	29
Glycerophospholipid metabolism	ko00564	50
Vitamin digestion and absorption	ko04977	29
Regulation of lipolysis in adipocytes	ko04923	28
Necroptosis	ko04217	21
Neurotrophin signaling pathway	ko04722	8
Adipocytokine signaling pathway	ko04920	8
Sphingolipid metabolism	ko00600	25
AGE-RAGE signaling pathway in diabetic complications	ko04933	8
Leishmaniasis	ko05140	8
Glycerolipid metabolism	ko00561	33
Inositol phosphate metabolism	ko00562	5
Phosphatidylinositol signaling system	ko04070	5
Long-term depression	ko04730	5
Choline metabolism in cancer	ko05231	32
Arachidonic acid metabolism	ko00590	27
Linoleic acid metabolism	ko00591	27
alpha-Linolenic acid metabolism	ko00592	27
Retrograde endocannabinoid signaling	ko04723	40
Glycosylphosphatidylinositol (GPI)-anchor biosynthesis	ko00563	13
Autophagy—other	ko04136	13
Autophagy	ko04140	13
Pathogenic Escherichia coli infection	ko05130	13
Kaposi sarcoma-associated herpesvirus infection	ko05167	13
Thermogenesis	ko04714	28

**Table 11:** Nine KEGG enrichment pathways of lipidomics and proteomics.

Pathway	Number of Lipids	Number of Proteins	Differential Lipids	Differential Proteins (Up-Regulated)	Differential Proteins (Down-Regulated)
Themogenesis	27	2	TG	NPR-A, HRAs	
Fat digestion and absorption	27	2	TG	FABP1	ABCA1
Cholesterol metabolism	27	2	TG		ABCA1, VDCA1
Metabolic pathways	48	17	TG	NPR-A, FA-CoA, UGT	PI4Kβ, AK1R1D1, PEPCK, L2HGDH, CBS
Arachidonic acid metabolism	11	2	TG		CYP2U1
Inositol phosphate metabolism	3	2	TG	ITPK1	PI4Kβ
Phosphatidylinositol signaling system	3	2	TG	ITPK1	PI4Kβ
Long-term depression	3	2	TG	GNAQ, HRAs	
Sphingolipid signaling pathway	2	2	SM	GNAQ, HRAs	

## Data Availability

Data available on request.

## Supplementary Materials

The following supporting information can be downloaded at: https://www.mdpi.com/article/10.3390/metabo12080759/s1, Table S1: Protein accession.
